# A multi-cohort validated OXPHOS signature predicts survival and immune profiles in grade II/III glioma patients

**DOI:** 10.3389/fimmu.2025.1638824

**Published:** 2025-08-01

**Authors:** Jun Mou, Min Zhang, Fumin Qin, Yajie Cui, Keyou Xu, Baoye Pang, Xinyue Li, Wanyi Tan, Aiqi Yang, Yaxin Liu, Lingjun Shen, Yanting Liu, Kai Xu

**Affiliations:** ^1^ Laboratory of Infectious Diseases and Vaccine, West China Hospital, West China School of Medicine, Sichuan University, Chengdu, China; ^2^ Cancer Research Institute, Cancer Hospital, The First Affiliated Hospital of Xinxiang Medical University, Weihui, China; ^3^ West China School of Public Health and West China Fourth Hospital, Sichuan University, Chengdu, Sichuan, China

**Keywords:** grade II/III gliomas, mitochondrial oxidative phosphorylation, prognostic gene signature, RiskScore model, immune microenvironment

## Abstract

**Introduction:**

Grade II/III gliomas are invasive brain tumors with a high risk of malignant progression and significant clinical heterogeneity, highlighting the urgent need for reliable prognostic biomarkers to guide personalized treatment strategies. This study aimed to investigate the molecular mechanisms driving glioma progression and to identify potential therapeutic targets.

**Methods:**

We analyzed 200 mitochondrial oxidative phosphorylation (OXPHOS)-related genes in 512 grade II/III glioma samples from The Cancer Genome Atlas (TCGA). Consensus clustering identified two distinct molecular subtypes (C1 and C2). Differentially expressed genes (DEGs) between subtypes were determined using the limma package. The immune cell composition and tumor microenvironment (TME) characteristics were assessed using ESTIMATE, MCPcounter, and CIBERSORT algorithms. Based on prognostic DEGs, we constructed a four-gene prognostic signature (MAOB, IGFBP2, SERPINA1, and LGR6).

**Results:**

The C2 molecular subtype was associated with poorer prognosis, higher immune scores, and enrichment in tumor-promoting pathways. The four-gene signature demonstrated strong prognostic performance and robustness across multiple independent validation cohorts. Immunohistochemical (IHC) analysis of clinical glioma specimens confirmed elevated protein expression levels of the four genes in tumor tissues.

**Discussion:**

Our OXPHOS-associated gene signature provides novel insights into the molecular classification, immune landscape, and prognosis of grade II/III gliomas. These findings lay the foundation for precision oncology and the development of targeted therapeutic interventions.

## 1 Introduction

Gliomas, which are originating from native glial cells (i.e., oligodendrocytes, astrocytes, and ependymal cells), exhibit histological features resembling their normal counterparts and represent the predominant primary central nervous system (CNS) malignancies (1). Characterized by high invasiveness, disability rates, recurrence rates, and mortality, gliomas are recognized as a formidable challenge in oncology, imposing substantial burdens on patients, families, and society (2, 3). Epidemiological data in China indicate an annual incidence of 5–8 per 100,000, accounting for approximately 40% of all intracranial tumors and 80% of malignant brain tumors, with 5-year mortality ranking third among systemic malignancies (3, 4). Current first-line therapy involves maximal safe surgical resection, yet even when combined with standard radiotherapy plus temozolomide or nitrosourea-based chemotherapy, the 5-year survival rate remains below 35%, underscoring poor prognostic outcomes (4, 5). The WHO(World Health Organization) classifies gliomas into grades I–IV based on histopathological and morphological criteria (6): Grade I as low-grade gliomas (LGGs), Grades II/III as anaplastic gliomas, and Grade IV as glioblastoma (GBM), with escalating malignancy at higher grades. Compared to GBM, Grades II/III gliomas exhibit lower malignancy, slower progression, and better prognoses (7). This study focuses on Grades II/III gliomas to establish molecular classification and prognostic biomarkers, thereby providing a foundation for precision therapy. Multidimensional characterization of the tumor microenvironment (TME) may reveal critical therapeutic targets, facilitating innovative strategies to improve patient survival.

Cellular metabolic regulation hinges on the dynamic interplay between mitochondrial oxidative phosphorylation (OXPHOS) and glycolysis, two ATP-generating pathways whose imbalance profoundly influences cell fate (8). Tumor metabolism serves as a pivotal link between the TME and malignant progression. While classical theories emphasize the Warburg effect—enhanced aerobic glycolysis and lactate production despite oxygen availability, coupled with OXPHOS suppression (9)—recent studies challenge this paradigm. Emerging evidence highlights OXPHOS as a key driver of malignancy in specific cancers through metabolic adaptability, underscoring its mechanistic complexity in tumor biology (10–12). Tissue-specific OXPHOS regulation is evident: prostate cancer exploits upregulated OXPHOS complexes for survival advantage (13); colorectal cancer leverages elevated OXPHOS to fuel proliferation, stemness, and chemoresistance (14–16); and cholangiocarcinoma (CCA) versus hepatocellular carcinoma (HCC) studies reveal organ-specific roles—OXPHOS sustains CCA stemness, whereas its hyperactivation in HCC paradoxically inhibits proliferation (17, 18). Notably, metabolic crosstalk within the TME is critical; for instance, inhibiting OXPHOS in cancer-associated fibroblasts (CAFs) suppresses oral squamous cell carcinoma progression (19).

In gliomas, metabolic heterogeneity manifests as glycolytic dominance in the tumor core versus OXPHOS dependency in invasive fronts and glioma stem cells (GSCs) (20). Glioblastoma upregulates OXPHOS to enhance survival, while GSC reliance on OXPHOS drives radio-/chemoresistance, with OXPHOS inhibition sensitizing tumors to therapy (21–23). Crucially, progressive upregulation of OXPHOS-related genes during malignant transformation from LGGs to Grades II/III gliomas and ultimately GBM highlights its critical role in glioma progression (24). Thus, delineating OXPHOS-associated molecular subtypes in Grades II/III gliomas holds significant prognostic and therapeutic implications, enabling targeted OXPHOS inhibition to improve clinical outcomes.

In this study, we established two molecular subtypes of Grades II/III gliomas based on mitochondrial OXPHOS-related genes and evaluated their associations with prognosis and clinical features. By identifying differentially expressed genes (DEGs) between subtypes, we constructed a four-gene prognostic signature (*MAOB, IGFBP2, SERPINA1*, and *LGR6*) and validated its robustness using the Chinese Glioma Genome Atlas (CGGA) expression dataset. This signature demonstrated superior performance in prognostic stratification and emerged as a critical tool for identifying novel therapeutic targets. Our findings provide pivotal insights into the pathogenesis, prognostic classification, and clinical management of Grades II/III gliomas, advancing precision oncology strategies.

## 2 Results

### 2.1 Non-negative matrix factorization-based molecular subtyping

**Figure 1:**
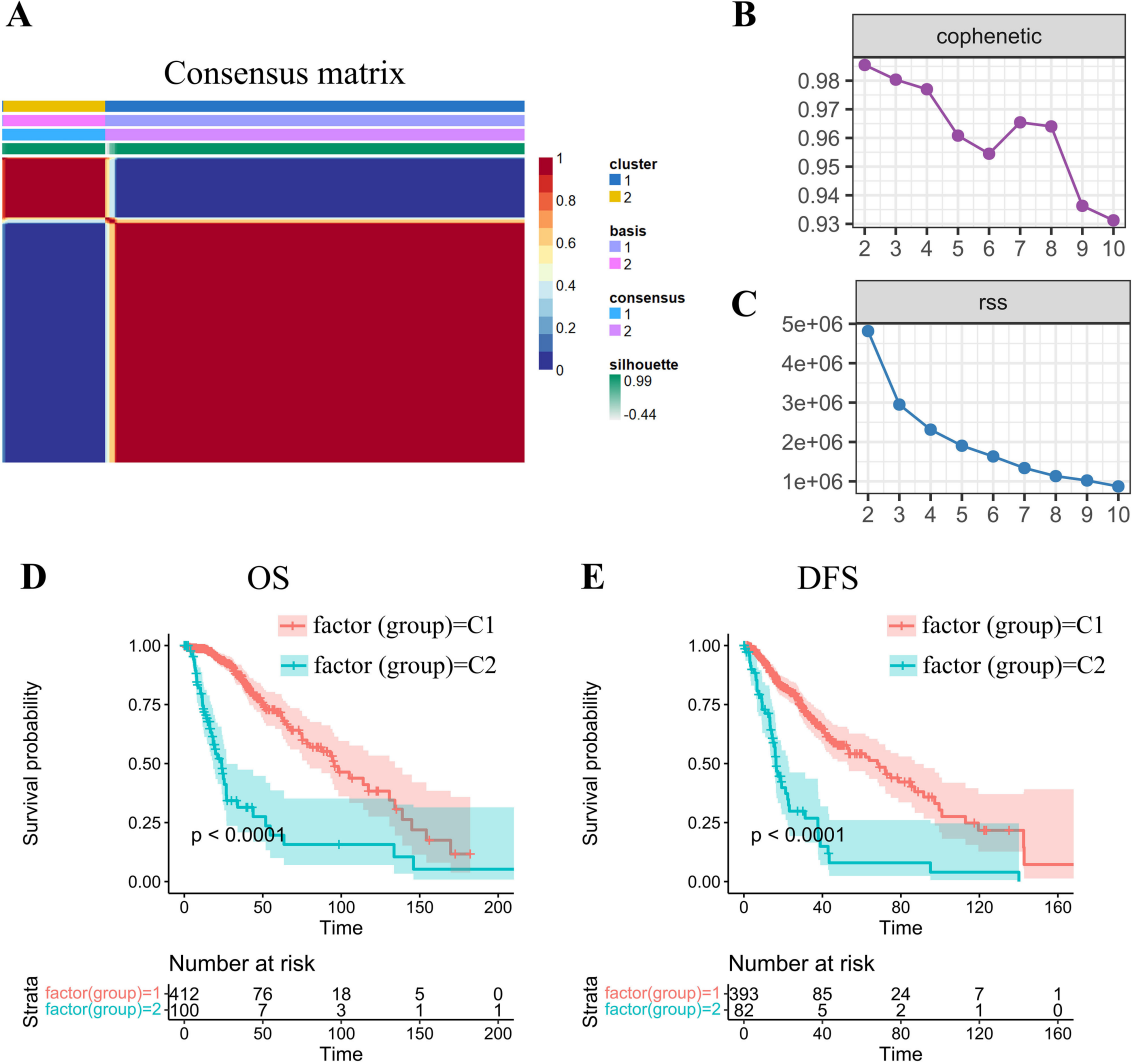
NMF-driven stratification and prognostic profiling of -gradeII/III gliomas through molecular subtype. **(A)** Consensus map of NMF clustering demonstrating subtype stability patterns. **(B)** Cophenetic correlation coefficient distribution across factorization ranks (*k=2-10*), calculated per Brunet’s methodology to assess clustering reproducibility. Higher values (approaching 1.0) indicate more robust subtype configurations. **(C)** Residual sum of squares (RSS) analysis demonstrating model fitting efficiency. Optimal clustering corresponds to the inflection point where RSS reduction plateaus. **(D, E)** Kaplan-Meier survival curves revealing significant prognostic divergence between molecular subtypes for both **(D)** overall survival (OS) and **(E)** disease-free survival (DFS) endpoints.

Using gene transcriptional profiles of grade II/III gliomas from TCGA, we extracted expression data of 200 OXPHOS-related genes. Univariate Cox analysis identified 77 statistically significant prognostic genes in grade II/III gliomas (*p* < 0.05; **Supplementary Table S1**). Utilizing expression patterns of these 77 prognostic genes, we implemented the NMF algorithm to cluster glioma samples. Through multimodal evaluation of consensus distributions, residual sum of squares (RSS), and other metrics, we identified *k=2* as the optimal cluster number, yielding two biologically distinct molecular subtypes (C1/C2; **Figures 1A–C**).

Subsequent prognostic analysis revealed significant clinical differences between the C1 and C2 subtypes, with both overall survival (OS) and disease-free survival (DFS) (log-rank *p* < 0.01; **Figures 1D, E**). Patients stratified into the C1 molecular subtype demonstrated a more favorable prognosis than those in the C2 subgroup.

### 2.2 Immune and stromal landscape differences between molecular subtypes

**Figure 2:**
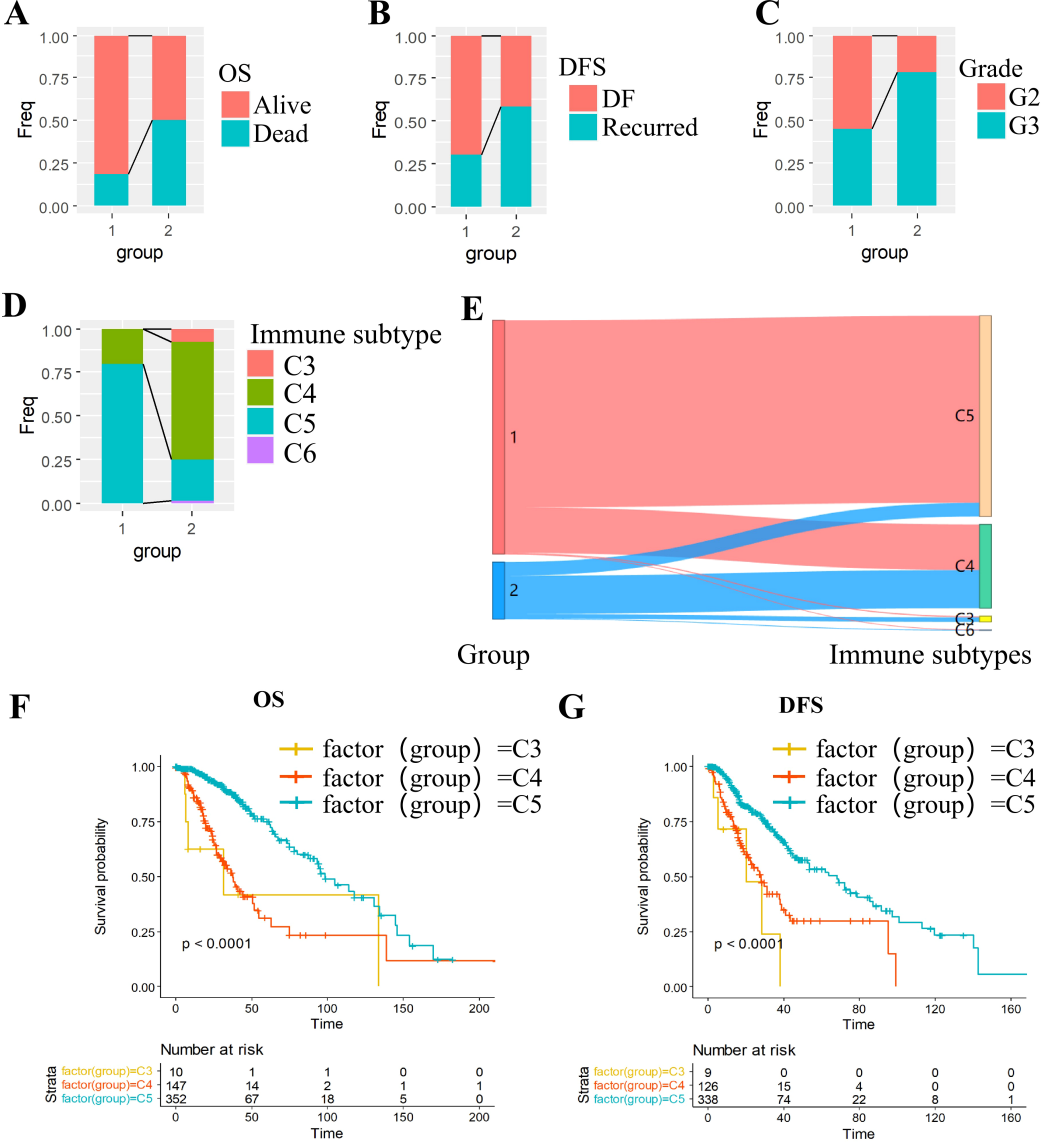
Multidimensional characterization of molecular subtypes in grade II/III gliomas. **(A-C)** Clinical feature stratification: **(A)** OS, **(B)** DFS and **(C)** grade. **(D-E)** Immune landscape: **(D)** Immune subtype distribution across molecular subtypes; **(E)** Concordance with established immune classifications. **(F, G)** Survival outcomes: **(F)** Overall survival by immune subtype; **(G)** Disease-free survival by immune subtype.

To delineate clinical heterogeneity between subtypes, we compared survival rates, recurrence proportions, and grade distributions. The C2 subtype exhibited significantly inferior survival outcomes compared to C1 (**Figure 2A**). Notably, the C2 subgroup demonstrated a higher tumor recurrence rate (**Figure 2B**) and an overrepresentation of prognostically unfavorable Grade 3 (G3) tumors (**Figure 2C**).

We cross-referenced our subtypes with the six established pan-cancer immune infiltrate subtypes: C1 (Wound Healing), C2 (IFN-γ Dominant), C3 (Inflammatory), C4 (Lymphocyte-Depleted),C5 (Immunologically Quiet),C6 (TGF-β Dominant) (25). Kaplan-Meier analysis revealed poorer prognosis in immunophenotypes C3 and C4 compared to C5 (**Figures 2F, G**). Intersectional analysis with our OXPHOS-based subtyping demonstrated significant enrichment of high-risk immunophenotypes (C3/C4) within the OXPHOS-C2 subgroup (**Figures 2D, E**), suggesting a mechanistic link between OXPHOS dysregulation and immunosuppressive tumor microenvironments.

### 2.3 Comparative immune score between molecular subtypes

To comprehensively characterize the immune landscape disparities between molecular subtypes, we employed three complementary computational approaches using TCGA datasets: (1) stromal, immune, and ESTIMATE scores via the ESTIMATE(Infers global immune/stromal content from bulk transcriptomes) R package; (2) MCP-counter quantifying 10 cellular populations (8 immune cells and 2 stromal cells); and (3) CIBERSORT deconvolution of 22 tumor-infiltrating immune cell subtypes, encompassing adaptive/innate immune lineages and functional states.

**Figure 3:**
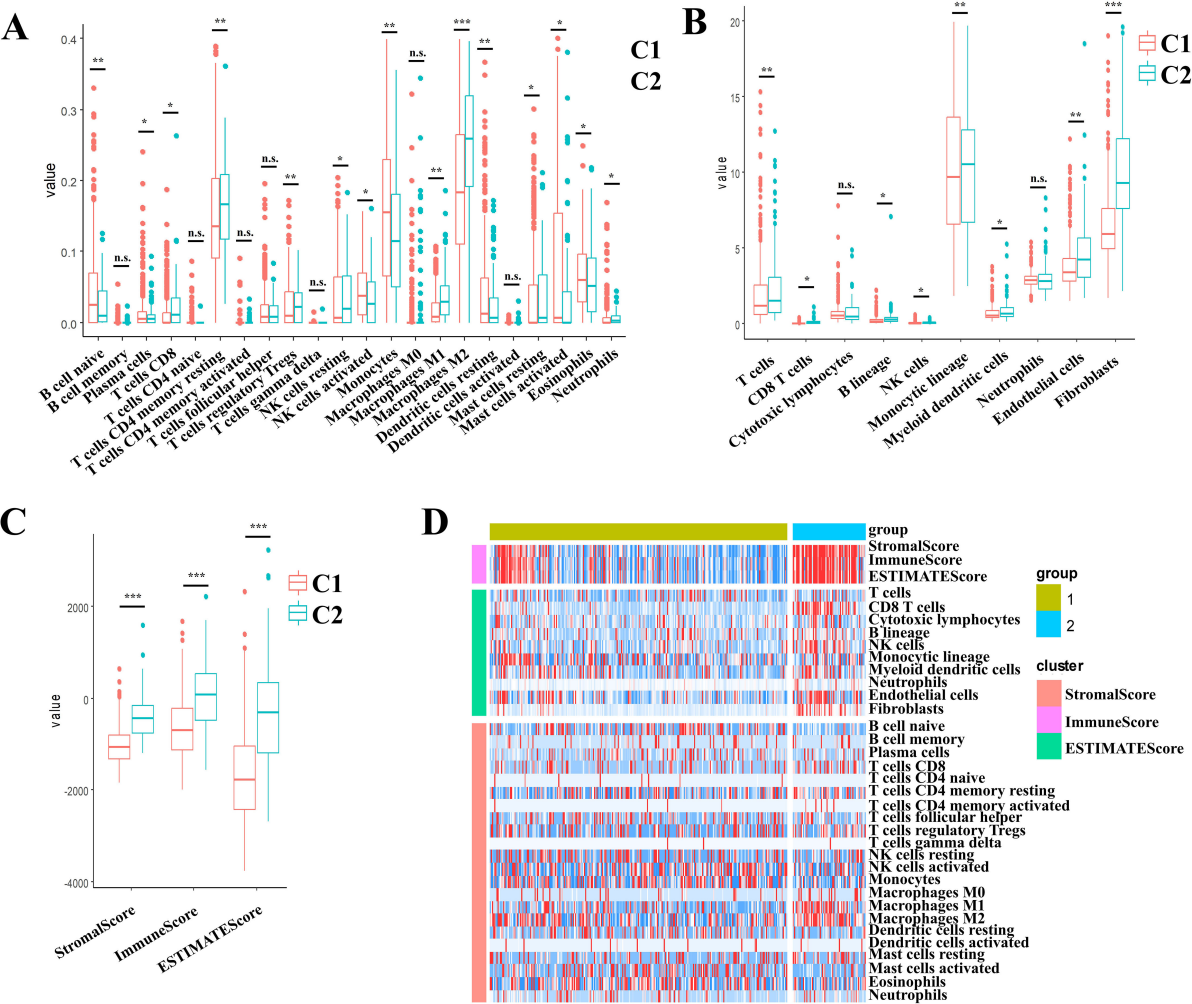
Multidimensional evaluation of immune landscape characterization in II/III gliomas through multi-algorithm deconvolution of TCGA data set. **(A)** CIBERSORTx-derived patterns across molecular subtypes. **(B)** MCP-counter quantified stratification across the molecular subtypes. **(C)** ESTIMATE-based distribution across the molecular subtypes. **(D)** Consensus heatmap of multi-algorithm immune signatures across the molecular subtypes. Boxplots indicate median immune scores with interquartile ranges (IQR). * *p* < 0.05, ** *p* < 0.01, *** *p* < 0.001, ns, not significant (*p* ≥ 0.05).

Comparative evaluation demonstrated that the C2 subtype displayed markedly elevated immune scores across all metrics. This signature corresponds to an immunosuppressive microenvironment featuring M2 macrophages dominance and stromal activation, accounting for its poor prognosis despite high immune scores (**Figures 3A-C**). Heatmap visualization demonstrated C2’s distinct immunoprofile dominated by immunosuppressive elements (M2 macrophages) and stromal activation signatures (**Figure 3D**). This paradoxical association - where elevated immune scores correlate with poorer prognosis in C2 - aligns with emerging paradigms of non-inflammatory immune infiltration driving therapeutic resistance in gliomas.

### 2.4 Differentially expressed genes analysis across subtypes

**Figure 4:**
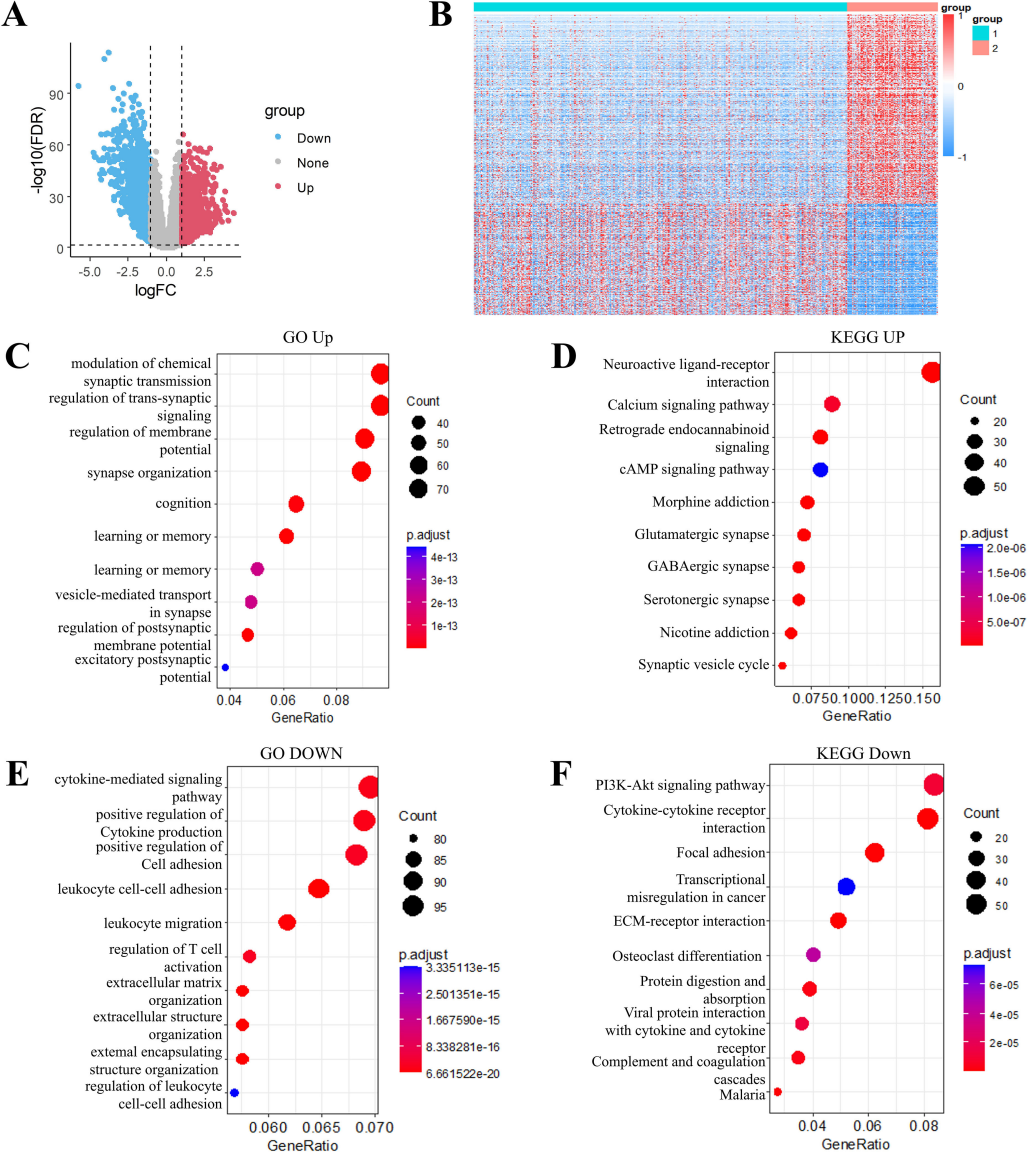
Transcriptomic landscape and functional annotation of molecular subtypes in grade II/III gliomas. **(A)** Volcano plot of differentially expressed genes between subtype C1 and C2. **(B)** Heatmap of differential genes between subtype C1 and C2. **(C)** Gene Ontology (GO) biological processes enriched in up-regulated genes. **(D)** KEGG pathway analysis of up-regulated gene signatures. **(E)** GO biological processes associated with down-regulated genes. **(F)** KEGG pathway mapping of down-regulated gene sets.

Differential gene expression analysis was performed using the limma package to compare transcriptional profiles across the C1 and C2 molecular subtypes. Applying stringent criteria including FDR < 0.01 and |log2FC| > 1, we identified 2535 significantly DEGs, comprising 933 upregulated and 1602 downregulated genes in the comparison (**Supplementary Table S2**). Comparative analysis displayed predominantly upregulated DEGs expression in the C2 subtype compared to C1 subtype. (**Figure 4A**). All DEGs were selected to generate a heatmap (**Figure 4B**).

Through the use of Goplot in R package, GO functional enrichment analysis of the 933 upregulated DEGs in the C2 molecular subtype of grade II/III gliomas revealed significant associations with pathways such as modulation of chemical synaptic transmission, regulation of trans-synaptic signaling, regulation of membrane potential, and synapse organization (**Figure 4C**). KEGG pathway enrichment analysis of these upregulated DEGs highlighted pathways including neuroactive ligand-receptor interaction, calcium signaling pathway and cAMP signaling pathway, (**Figure 4D**). Similarly, functional enrichment analysis of the 1602 downregulated DEGs was carried out by using GO and KEGG. The downregulated DEGs in the C2 subtype were primarily enriched for pathways such as PI3K-Akt signaling pathway, cytokine-cytokine receptor interaction, focal adhesion, and transcriptional misregulation in cancer (**Figures 4E, F**).

### 2.5 Construction of prognostic risk models

**Figure 5:**
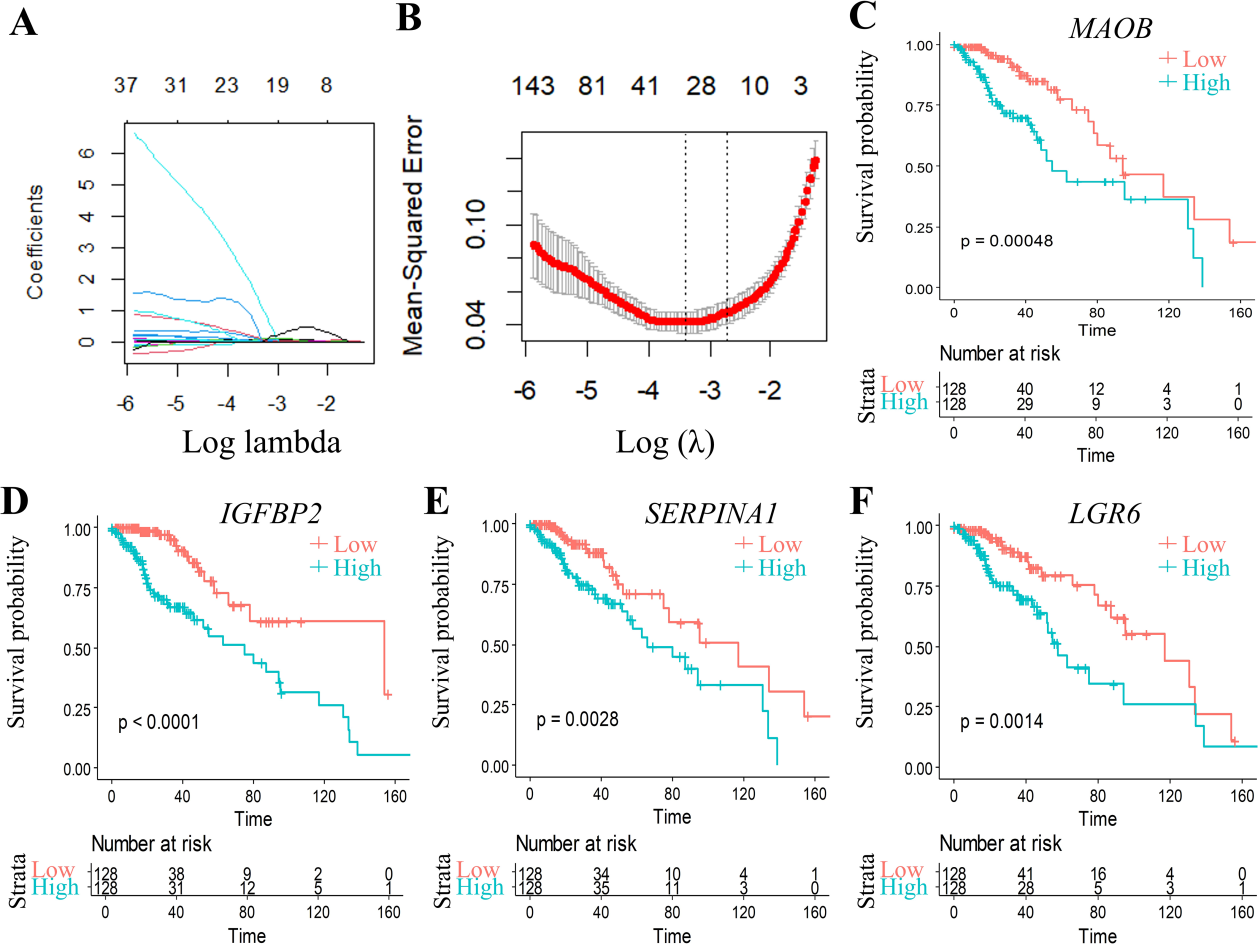
Multivariable analysis with confidence interval estimation and survival correlation of molecular features in the TCGA training set. **(A)** LASSO coefficient trajectories: x-axis (log(λ)), y-axis (coefficient estimates). **(B)** Cross-validated confidence intervals for λ selection. **(C-F)** KM curves for 4 genes signature (TCGA training set). *p < 0.05, **p < 0.01, ***p < 0.001, ns, not significant (p ≥ 0.05).

To further build the RiskScore model(Patients were stratified into high-risk and low-risk groups using the median RiskScore as the cutoff threshold.), we randomly divided data from 512 patients into training (n = 256) and validation cohorts (n = 256). Initially, 2166 DEGs were detected when comparing C1 and C2 subtypes. Univariate Cox proportional hazards regression analysis of survival data (*p* < 0.01) identified 874 prognosis associated genes (**Supplementary Table S3**). To enhance clinical utility, LASSO regression was conducted with the glmnet in R to reduce gene dimensionality. The trajectory of variable coefficients with increasing lambda values is shown in **Figure 5A**. Ten-fold cross-validation (**Figure 5B**) determined the optimal lambda, yielding 48 candidate genes.

Subsequent stepwise regression supported by the Akaike Information Criterion (AIC) via the MASS package refined these 48 genes to four core biomarkers: *MAOB, IGFBP2, SERPINA1*, and *LGR6*. Prognostic Kaplan-Meier curves (**Figures 5C–F**) demonstrated significant stratification (*p* < 0.05) in training set.

**Figure 6:**
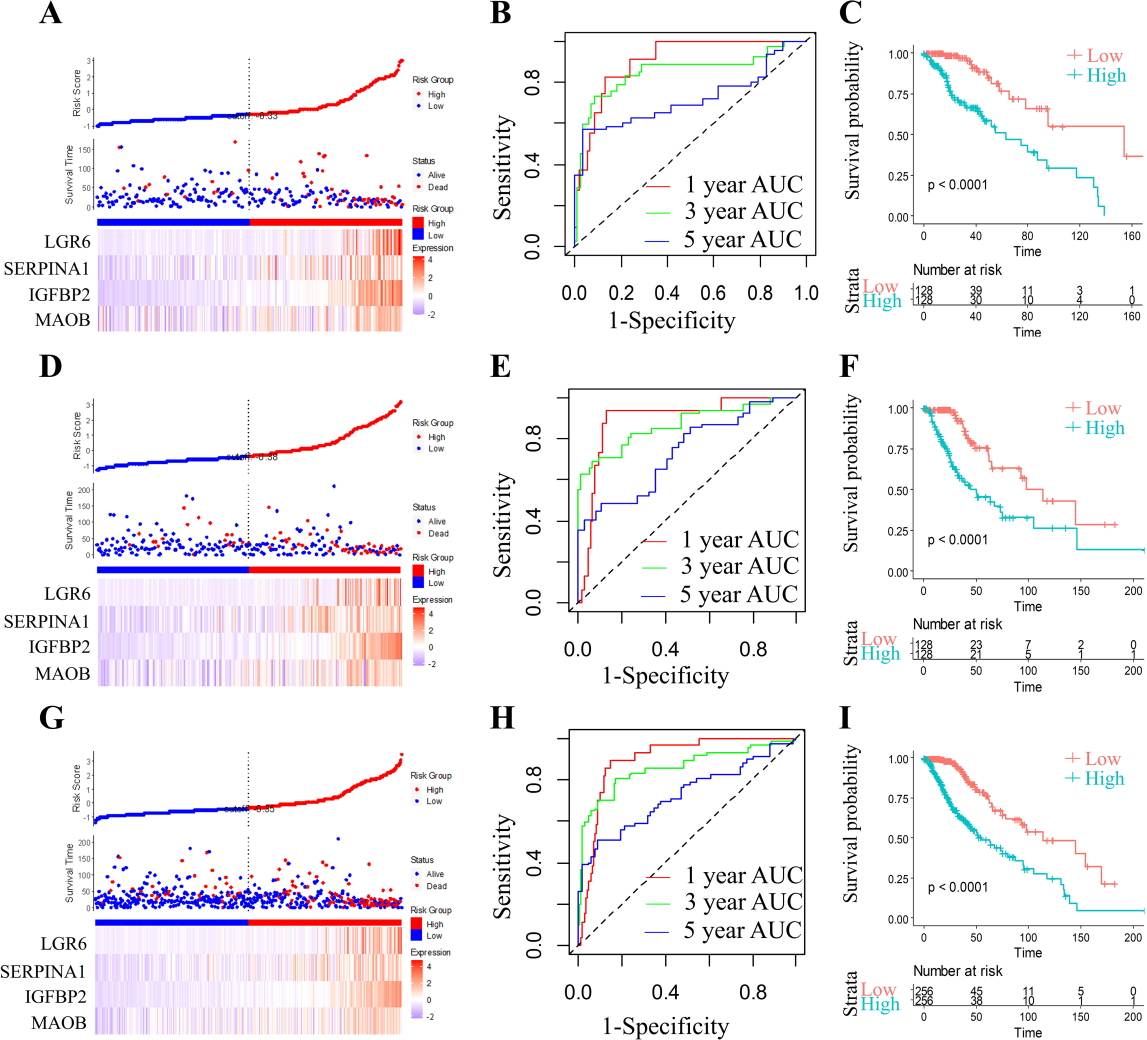
Comprehensive prognostic model evaluation: Risk score, gene expression, ROC, and survival distribution in TCGA training, test, and full TCGA datasets. Risk score, survival status, and 4 gene expression in the TCGA training set **(A)**, the TCGA test set **(D)**, and the full TCGA dataset **(G)**. ROC curves and AUCs for the 4 gene features in the TCGA training set (**B**, 1 year AUC=90.44,95% CI 0.839-0.97; 3 year AUC=84.77,95% CI 0.745-0.95; 5 year AUC=71.82,95% CI 0.578-0.859), the TCGA test set (**E**, 1 year AUC=89.35,95% CI 0.817-0.969; 3 year AUC=85.58,95% CI 0.783-0.948; 5 year AUC=73.24,95% CI 0.627-0.837), and the full TCGA dataset (**H**, 1 year AUC=89.62,95% CI 0.849-0.942; 3 year AUC=85.68,95% CI 0.793-0.92; 5 year AUC=72.47,95% CI 0.639-0.81). Distribution of KM survival curves for the 4 gene features in the TCGA training set **(C)**, the TCGA test set **(F)**, and the full TCGA dataset **(I)**.

RiskScore for each sample was calculated using the ggRISK package, with lower scores indicating better prognosis (**Figure 6A**). Time-dependent receiver operating characteristic (ROC) analysis (timeROC package) showed AUC values exceeding 0.71 for 1-, 3-, and 5-year survival predictions (**Figure 6B**). Finally, Kaplan-Meier analysis stratified by RiskScore thresholds confirmed significantly superior survival outcomes in the low-risk group (**Figure 6C**).

### 2.6 Validation of the risk models

In order to assess the model stability, we applied the identical RiskScore model and its pre-defined coefficients (derived from the training cohort) to validate the TCGA datasets. As illustrated, the RiskScore distribution within the TCGA validation cohort exhibited a dose-dependent relationship between increasing RiskScore values and worsening prognosis in grade II/III glioma patients (**Figure 6D**). ROC analysis further quantified the predictive accuracy, achieving area under the curve (AUC) values surpassing 0.73 for 1-, 3-, and 5-year survival outcomes (**Figure 6E**). Strikingly, Kaplan-Meier survival curves demonstrated a profound divergence between high- and low-RiskScore groups (**Figure 6F**; *p* < 0.0001), underscoring the model’s discriminative power.

To ensure generalizability, we expanded the validation to the full TCGA cohort. The RiskScore distribution (**Figure 6G**) consistently mirrored the trend observed in the validation subset, with elevated RiskScores correlating with adverse clinical outcomes. ROC analysis for the complete cohort (**Figure 6H**) maintained robust predictive performance, yielding AUC values > 0.72 across all time points. Kaplan-Meier analysis again confirmed statistically significant survival stratification (**Figure 6I**, p < 0.0001), with the low-RiskScore group exhibiting markedly prolonged survival.

### 2.7 External validation of the four-gene signature stability

**Figure 7:**
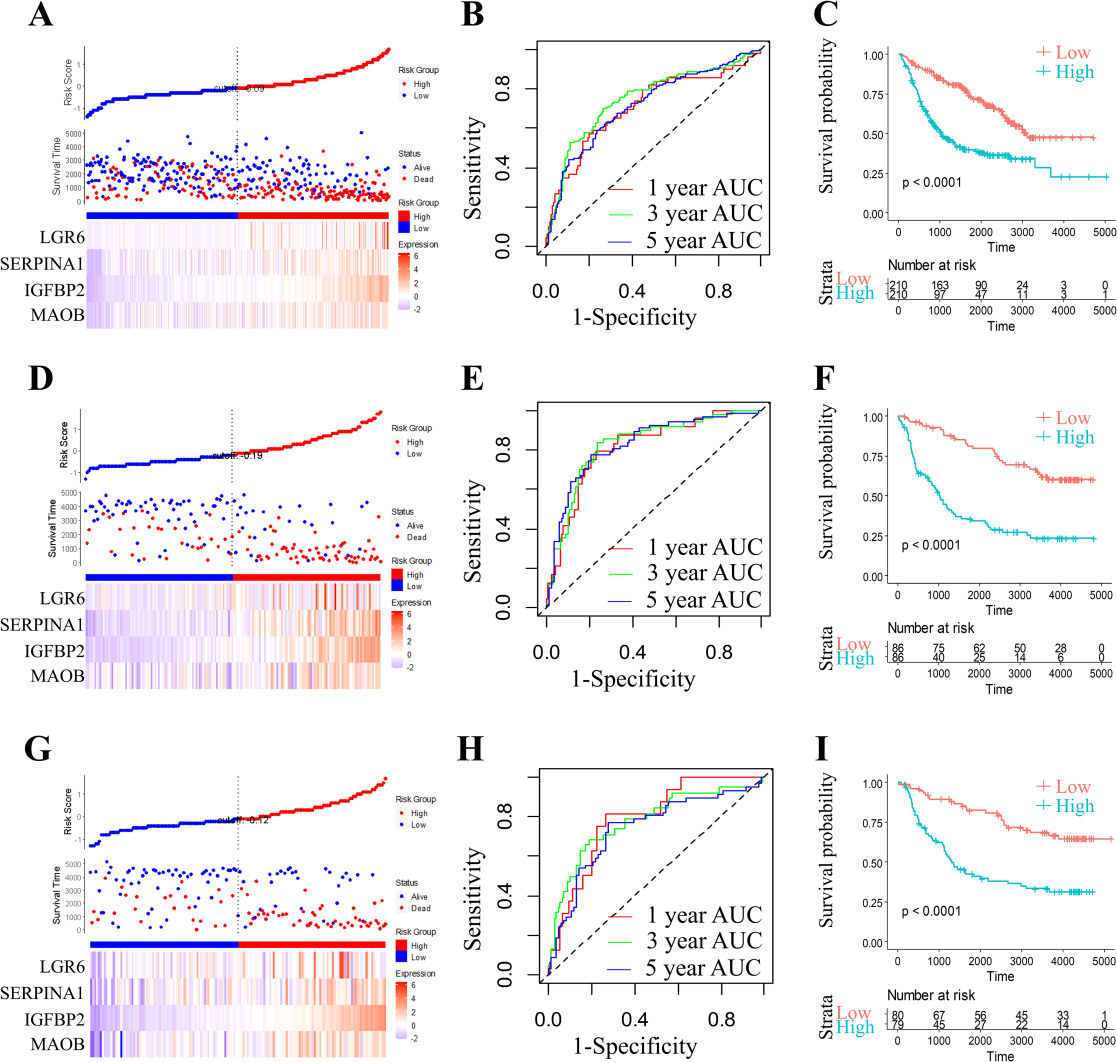
Comprehensive prognostic model evaluation: Risk score, gene expression, ROC, and survival distribution in three CGGA (CGGA-693, CGGA-325, and CGGA-301) datasets. Risk score, survival status, and 4 gene expression in the CGGA-693 **(A)**, the CGGA-325 **(D)**, and the CGGA-301 dataset **(G)**. ROC curves and AUCs for the 4 gene features in the CGGA-693 (**B**, 1 year AUC=71.01,95% CI 0.624-0.795; 3 year AUC=74.74,95% CI 0.693-0.803; 5 year AUC=71.62,95% CI 0.66-0.772), the CGGA-325 (**E**, 1 year AUC=81.25,95% CI 0.726-0.902; 3 year AUC=82.65,95% CI 0.756-0.896; 5 year AUC=82.65,95% CI 0.759-0.894), and the CGGA-301 dataset (**H**, 1 year AUC=79.11,95% CI 0.69-0.891; 3 year AUC=77.60,95% CI 0.682-0.869; 5 year AUC=73.79,95% CI 0.65-0.825). Distribution of KM survival curves for the 4 gene features in the CGGA-693 **(C)**, the CGGA-325 **(F)**, and the CGGA-301 dataset **(I)**.

To rigorously validate the generalizability and clinical utility of our four-gene prognostic signature (*MAOB, IGFBP2, SERPINA1*, and *LGR6*), we evaluated the RiskScore model across three independent cohorts from the CGGA datasets. Applying the pre-trained coefficients from the original model, RiskScores were computed for all samples using the ggRISK R package. RiskScore distributions across all CGGA cohorts (**Figures 7A, D, G**) revealed a consistent trend: patients with higher RiskScores exhibited significantly shorter median survival. Time-dependent ROC analysis demonstrated robust predictive accuracy, with AUC values ranging from 0.71 to 0.90 for 1-, 3-, and 5-year survival (**Figures 7B, E, H**). Kaplan-Meier analysis further confirmed statistically distinct survival outcomes between risk strata (**Figures 7C, F, I**). The model’s stability across ethnically diverse populations (TCGA and CGGA) underscores its potential as a reproducible tool for guiding adjuvant therapy decisions in grade II/III glioma management.

### 2.8 External validation via pan-cancer analysis

**Figure 8:**
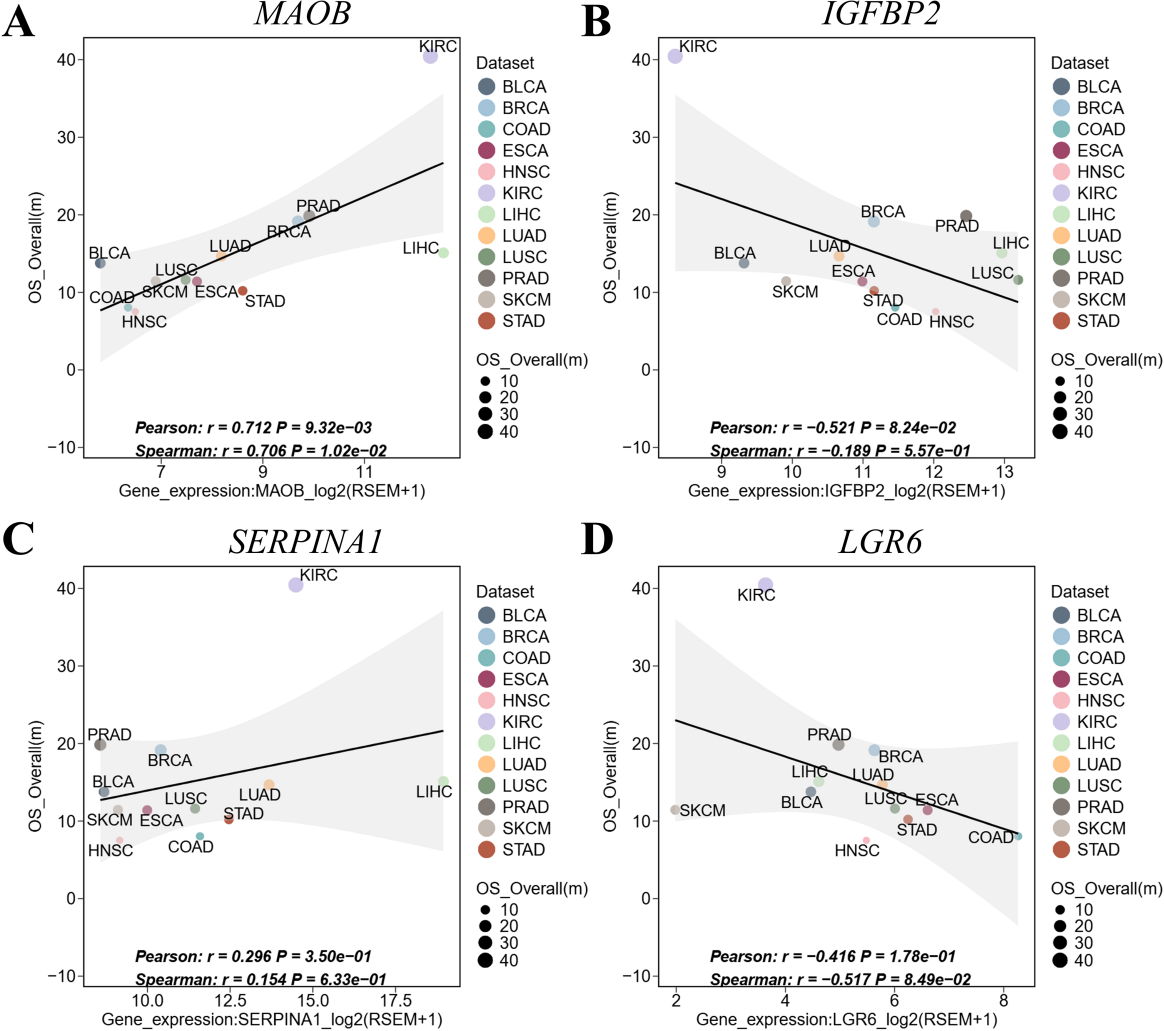
Pan-cancer validation of the 4-gene signature in ICBatlas. **(A)** *MAOB* expression vs. OS. **(B)** *IGFBP2* expression vs. OS. **(C)** *SERPINA1* expression vs. OS. **(D)** *LGR6* expression vs. OS. Each point represents a cancer type. Shaded areas: 95% CI of linear fit. Significance reflects cross-cancer consistency, not intra-tumor prognosis. BLCA, bladder urothelial carcinoma; BRCA, breast invasive carcinoma; COAD, colon adenocarcinoma; ESCA, esophageal carcinoma; HNSC, head and neck squamous cell carcinoma; HROS, hazard ratios for overall survival; KIRC, kidney renal clear cell carcinoma; LIHC, liver hepatocellular carcinoma; LUAD, lung adenocarcinoma; LUSC, lung squamous cell carcinoma; mOS, median overall survival; PRAD, prostate adenocarcinoma; SKCM, skin cutaneous melanoma; STAD, stomach adenocarcinoma.

Recent pan-cancer studies have demonstrated the value of multi-omics approaches in elucidating tumor immunity and therapeutic prognosis (26). To evaluate the pan-cancer robustness of our 4-gene signature (*MAOB, IGFBP2, SERPINA1* and *LGR6*), we leveraged the ICBatlas database—comprising 93,000+ patients across 18 cancer types (including 12 cancers with multi-omics profiling) and 30 immune checkpoint inhibitor (ICI) treatment cohorts. Key findings revealed divergent survival associations: *MAOB* (**Figure 8A**, r = 0.712, *P* = 9.32e-03) and *SERPINA1* (**Figure 8C**, r = 0.296, *P* = 3.50e-01) showed significant positive correlation with overall survival (OS), suggesting protective roles. While *IGFBP2* (**Figure 8B**, r =- 0.521, *P* = 8.24e-02) and *LGR6* (**Figure 8D**, r = -0.416, *P* = 1.78e-01) exhibited negative correlation with OS, indicating risk-enhancing effects. This bidirectional pattern positions the 4-gene signature as a pan-cancer prognostic framework, though tissue-specific validation remains essential.

### 2.9 Correlation analysis of the risk model with clinical features and biological pathways

**Figure 9:**
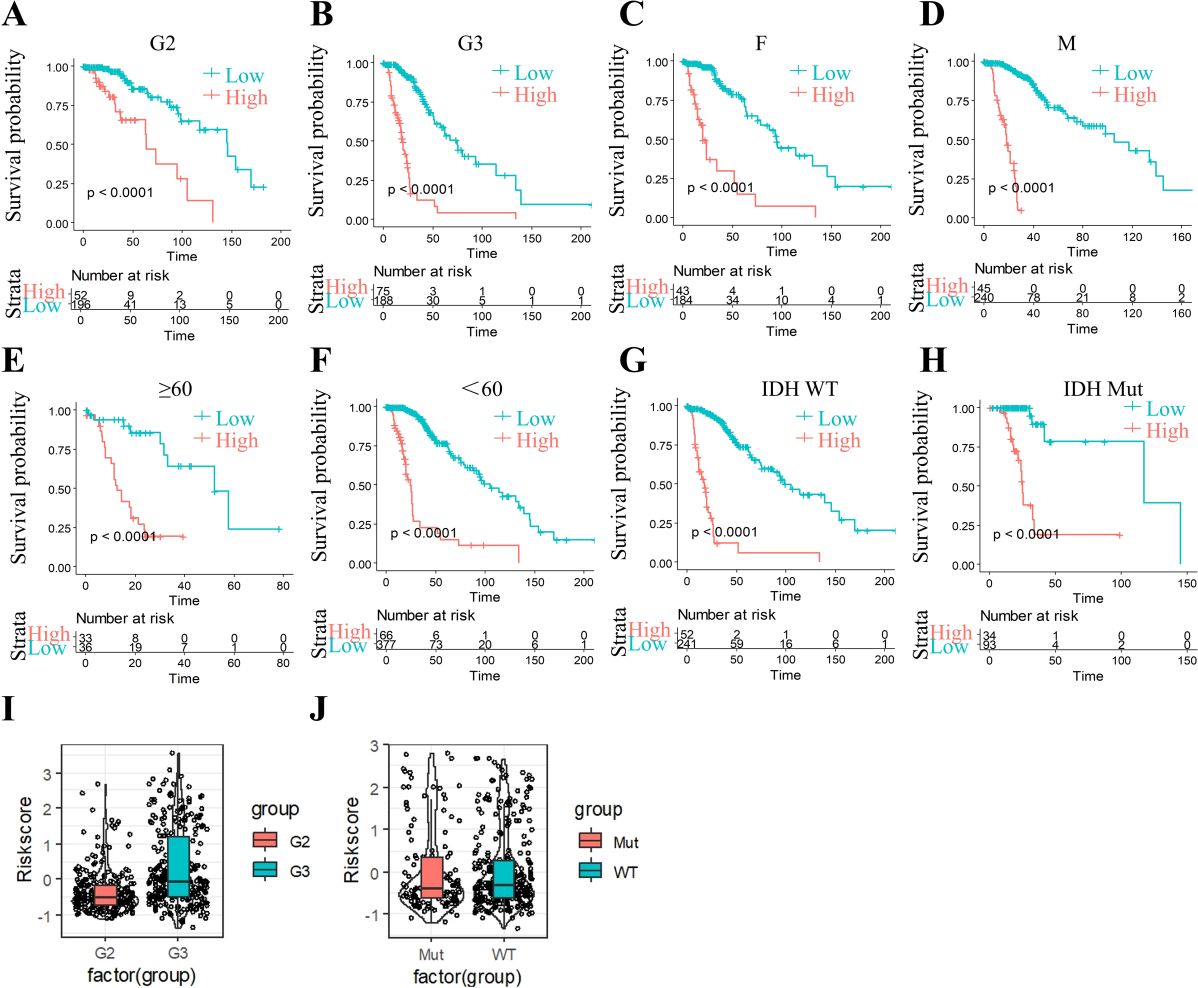
Riskscore stratification performance across clinical subgroups. **(A)** The significant prognosis according to Riskscore in group G2 patients. **(B)** The significant prognosis according to Riskscore in group G3 patients. **(C)** The significant prognosis according to Riskscore in female patients. **(D)** The significant prognosis according to Riskscore in male patients. **(E)** The significant prognosis according to Riskscore in age ≥60 patients. **(F)** The significant prognosis according to Riskscore in age <60 patients. **(G)** The significant prognosis according to Riskscore in IDH-WT patients. **(H)** The significant prognosis according to Riskscore in IDH-mutation patients. **(I)** Riskscore in G2 and G3. **(J)** Riskscore in IDH-WT group and IDH-mutation group.

The four-gene molecular signature demonstrated robust prognostic stratification across diverse clinical subgroups. Kaplan-Meier analysis revealed significantly worse survival outcomes in high-RiskScore groups compared to low-RiskScore groups within all grade-stage, gender, age, and IDH mutation subgroups (**Figures 9A–H** p < 0.05), underscoring the model’s generalizability. Notably, RiskScore distributions exhibited strong positive correlations with advancing G-stage (**Figures 9I, J**, *p* < 0.05), suggesting its intrinsic linkage to disease severity and further validating its prognostic utility.

**Figure 10:**
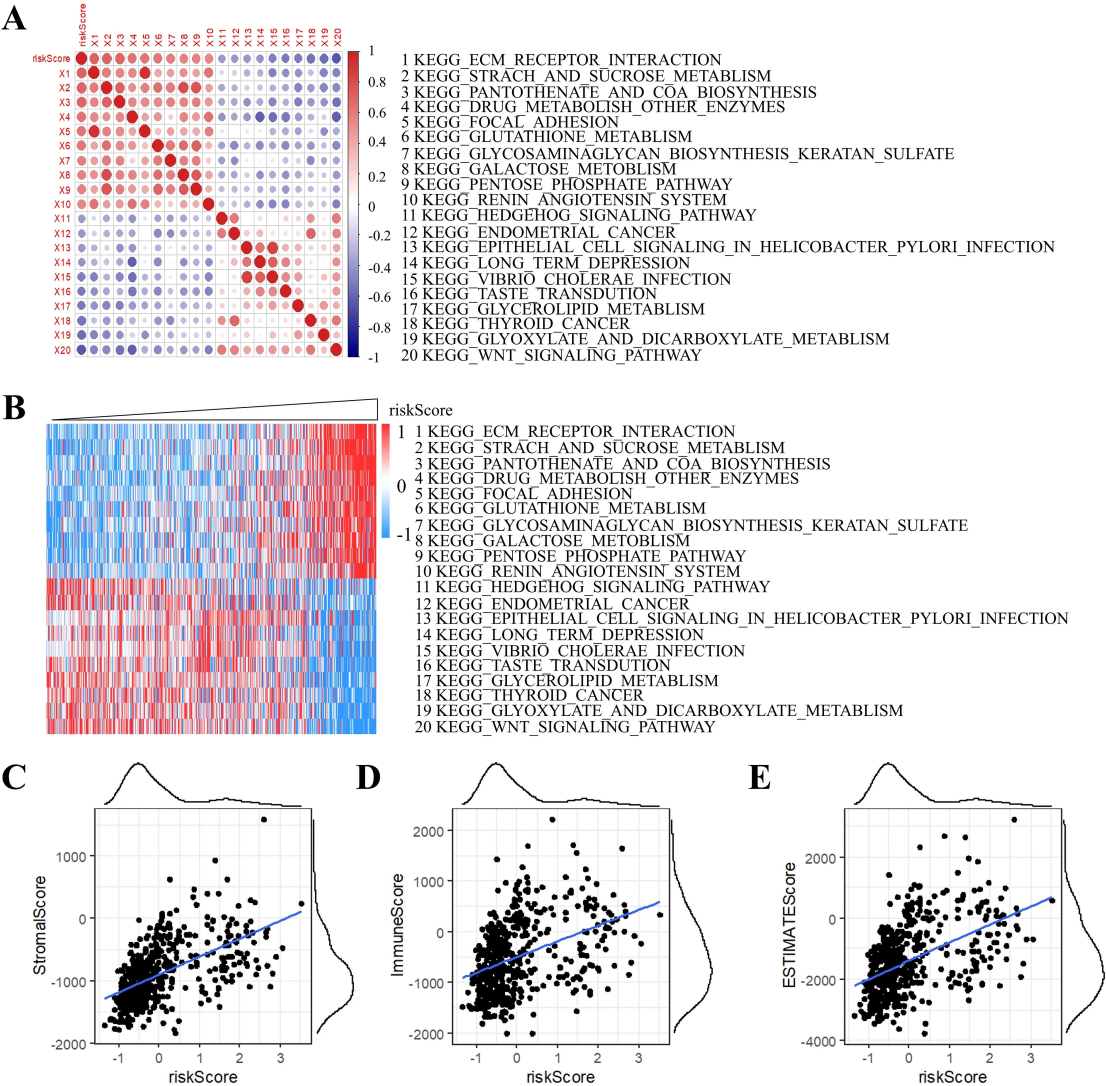
Correlation analysis of ssGSEA and RiskScore. **(A)** Correlation of RiskScore with ssGSEA KEGG pathway. **(B)** Heatmap visualization of ssGSEA KEGG pathways. **(C)** Stromal component association. **(D)** Immune infiltration correlation. **(E)** ESTIMATE-based tumor purity association.

To elucidate biological underpinnings, we performed GSEA pathway scoring. The top 10 KEGG pathways positively associated with RiskScore included ECM-receptor interaction, starch and sucrose metabolism, and pantothenate and CoA biosynthesis, while inversely correlated pathways encompassed Hedgehog signaling, endometrial cancer, and Wnt signaling (**Figures 10A, B**). These findings implicate RiskScore in extracellular matrix remodeling and metabolic reprogramming, hallmarks of grade II/III glioma progression.

Strikingly, RiskScore showed significant positive correlations with tumor microenvironment scores derived from the ESTIMATE algorithm: StromalScore, ImmuneScore, and ESTIMATEScore (**Figures 10C–E**), suggesting its dual role in predicting both tumor-intrinsic aggressiveness and immune microenvironment activation.

### 2.10 Multivariate Cox regression analyses of the four-gene signature

**Figure 11:**
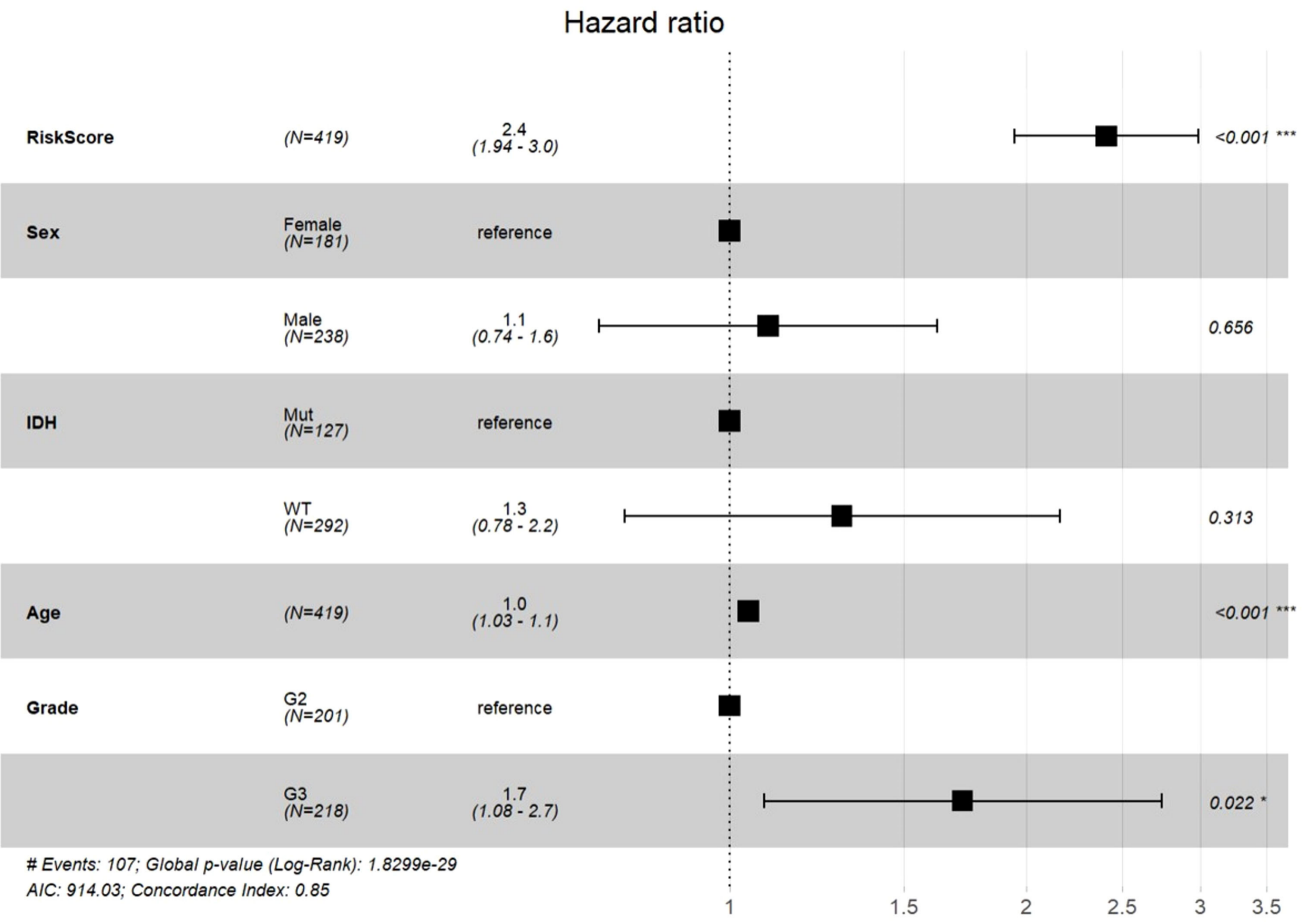
Multivariable Cox regression analysis with forest plot visualization of prognostic factors.

Finally, to validate the four-gene signature’s clinical independence in TCGA cohort, we conducted multivariate Cox regression analyses which demonstrated a significant association between RiskScore and overall survival (**Figure 11A**). These results underscore the robust clinical predictive performance of our four-gene signature (*MAOB, IGFBP2, SERPINA1*, and *LGR6*), establishing its capacity to independently prognosticate outcomes in cases with grade II/III gliomas, even after adjusting for age, grade stage, and IDH mutation status.

### 2.11 Immunohistochemical validation of the four-gene signature

**Figure 12:**
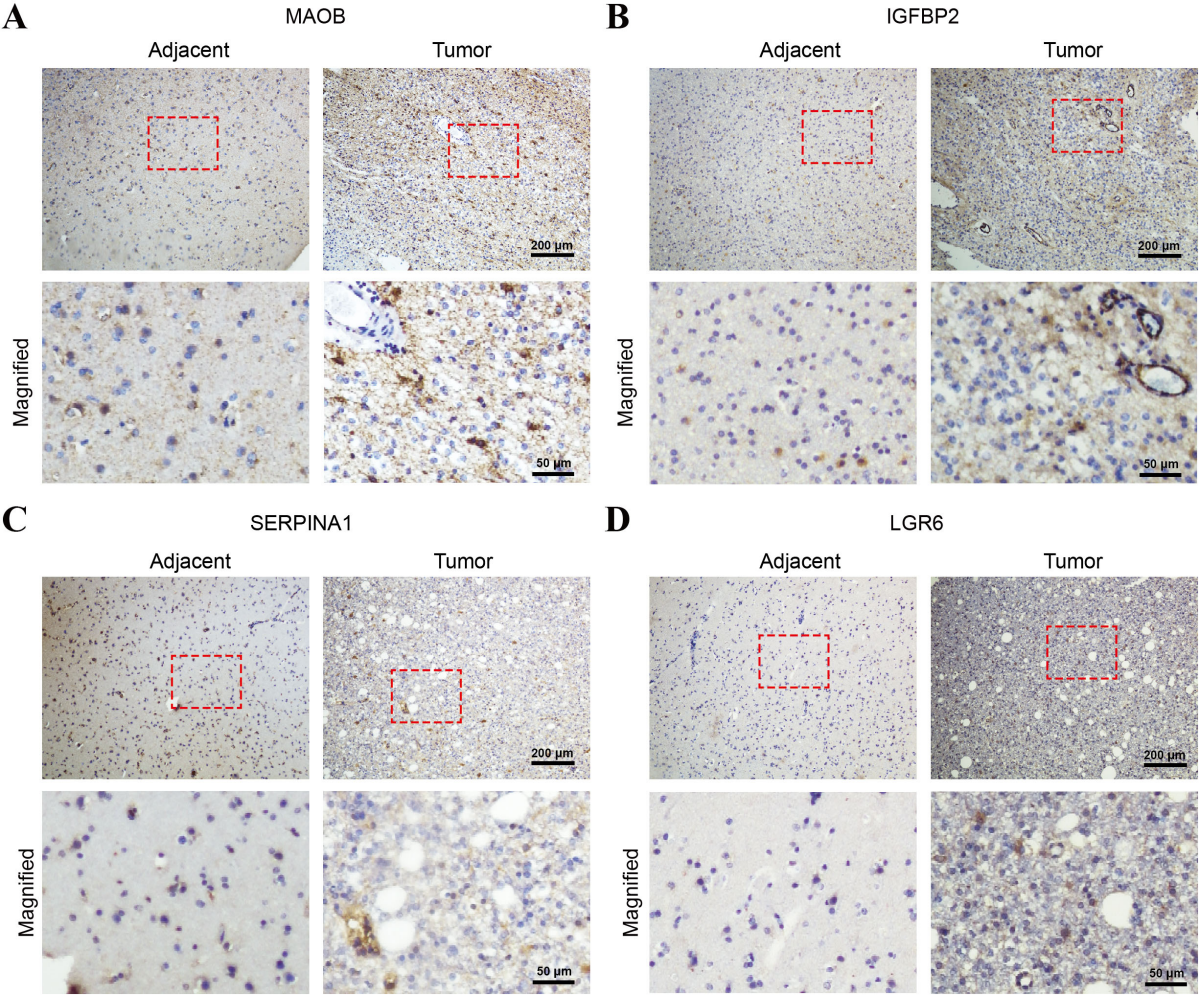
Immunohistochemical validation of the four-gene signature in glioma and adjacent non-tumorous brain tissues. Representative IHC staining images of **(A)** MAOB, **(B)** IGFBP2, **(C)** SERPINA1, and **(D)** LGR6 in paraffin-embedded glioma tissues and adjacent normal brain tissues. Each panel shows low-magnification images (top row; scale bar = 200 μm) and corresponding high-magnification views (bottom row; scale bars = 50 μm) of the selected areas (red boxes).

To validate the protein-level expression patterns of the prognostic genes in clinical specimens, we performed immunohistochemical (IHC) staining of MAOB, IGFBP2, SERPINA1, and LGR6 on paraffin-embedded sections of glioma tissues and adjacent non-tumorous brain tissues. All four proteins exhibit markedly increased expression in tumor tissues compared to adjacent tissues, supporting their transcriptomic upregulation and potential roles in glioma pathogenesis (**Figures 12A–D**).

## 3 Discussion

Grade II/III gliomas, as invasive tumors with malignant transformation potential, exhibit marked clinical heterogeneity, underscoring the critical need for prognostic biomarkers to guide personalized therapeutic strategies. Although grade II gliomas are classified as low-grade gliomas (LGGs), they carry inherent risks of recurrence and progression to higher-grade malignancies (e.g., grade III or IV), a process tightly linked to mitochondrial metabolic reprogramming (22). By analyzing 77 mitochondrial oxidative phosphorylation (OXPHOS)-related prognostic genes from the TCGA database across 512 grade II/III glioma samples, we identified two robust molecular subtypes, designated C1 and C2, which displayed significant differences in clinical characteristics and outcomes. Specifically, C2 patients exhibited poorer survival, higher mortality rates, advanced G-stage, and elevated immune scores compared to C1. The prognostic model developed herein not only distinguishes molecular subtypes but also provides novel insights into risk stratification for grade II/III gliomas.

While molecularly driven prognostic models are widely utilized for glioma classification, this study pioneers the subtyping of grade II/III gliomas depended on mitochondrial OXPHOS metabolic process. We established and validated a robust four-gene signature (*MAOB, IGFBP2, SERPINA1* and *LGR6*) that serve as an independent prognostic biomarker for grade II/III gliomas. Functional analyses revealed these genes participate in broad oncogenic processes, including proliferation, metastasis, and invasion, offering mechanistic insights into disease progression and therapeutic targeting. This discovery not only advances our understanding of glioma pathogenesis but also provides a theoretical foundation for developing novel treatment strategies.

Monoamine oxidase B(MAOB), a 520-amino acid mitochondrial outer membrane enzyme, catalyzes oxidative deamination of monoamines to regulate metabolism. While historically linked to neurodegenerative disorders, where MAOB deficiency contributes to neuronal dysfunction in Alzheimer’s disease (27–30). Emerging evidence reveals MAOB’s tissue-specific duality in oncology, exhibiting both pro- and anti-tumorigenic roles. In colorectal cancer, MAOB overexpression correlates with advanced staging, recurrence, and poor prognosis (31–33). In non-small cell lung cancer (NSCLC), MAOB serves as a diagnostic/prognostic marker, and its inhibition enhances radiosensitivity (34–36). Glioma studies show elevated MAOB activity in high-grade tumors drives ROS production, fostering a pro-tumorigenic microenvironment (37–39). Paradoxically, MAOB exhibits tumor-suppressive effects in endometrial cancer (40) and hepatocellular carcinoma (41), while its loss promotes renal (34, 42) and bladder cancer progression (43–45). Despite therapeutic potential of MAOB inhibitors (e.g., selegiline), inflammatory confounders necessitate further exploration of tissue-specific regulatory mechanisms (46).

Leucine-Rich Repeat-Containing G-Protein Coupled Receptor 6 (LGR6), a pivotal member of the G-protein coupled receptor (GPCR) superfamily (47), activates canonical Wnt/β-catenin signaling by binding R-spondin 1-3 (RSPO1-3) ligands [4 (48, 49). It plays core roles in maintaining the self-renewal and differentiation of stem cell populations, including skin hair follicle stem cells (50), taste bud progenitors (51), lung basal cells (52), and mammary epithelial stem cells (53). In oncology, LGR6 drives Wnt/β-catenin hyperactivation in ovarian cancer (54) and esophageal squamous cell carcinoma (ESCC) (55), facilitating metastasis and unfavorable prognosis. In triple-negative breast cancer (TNBC), LGR6 mediates stemness, chemoresistance, and metastasis (56). Non-Wnt pathways (e.g., PI3K/AKT/mTOR) contribute to its pro-survival roles in gastric (57), colorectal (58), and glioblastoma (GBM) (59), where it enhances temozolomide resistance. LGR6’s enrichment in advanced NSCLC and lung adenocarcinoma correlates with EMT and stem-like properties (60, 61), positioning it as a dual diagnostic/prognostic marker and druggable target.

Insulin like growth factor binding protein 2 (IGFBP2), a secreted regulatory protein and the second most abundant insulin-like growth factor binding protein (IGFBP) in circulation, bidirectionally modulates the bioactivity of IGFs through direct binding (62–64). Highly expressed in the hepatic tissue, adipocytes, and central nervous system, IGFBP2 plays pivotal roles in tumor growth, metabolism, and progression, positioning it as a potential biomarker across multiple cancers (65). In both pancreatic ductal adenocarcinoma (PDAC) and hepatocellular carcinoma (HCC), IGFBP2 induces epithelial-mesenchymal transition (EMT) by activating the NF-κB signaling pathway (66, 67). In metastatic breast cancer, tumor-secreted IGFBP2 recruits endothelial cells—a hallmark of metastatic dissemination (68)—while synergizing with β-catenin to promote lymph node metastasis (69, 70). Elevated plasma IGFBP2 levels correlate with malignancy risk in prostate cancer (71, 72) NSCLC studies link IGFBP2 overexpression to gefitinib resistance, reduced survival, and increased metastasis (73–75). In gliomas, IGFBP2 exhibits grade-dependent expression: mRNA overexpression in glioblastoma versus normal tissues predicts poor prognosis (76), while elevated protein levels in blood/tissue positively correlate with tumor grade (77, 78). Notably, high antibody titers in early detection distinguish grade II/III gliomas from healthy controls (79). Future research must elucidate tissue-specific regulatory networks and advance IGFBP2-driven liquid biopsy technologies and targeted therapies to propel precision oncology forward.

The Serpin Family A Member 1 (SERPINA1) gene, cloned in 1981 (80), encodes α1-antitrypsin (AAT), an acute-phase reactant and the predominant serine protease inhibitor in human plasma (81, 82). While predominantly expressed in hepatocytes, AAT is also synthesized in monocytes, neutrophils, and bronchial epithelial cells (83, 84). AAT maintains systemic homeostasis through four core mechanisms: (1) direct inhibition of protease activity, (2) modulation of cytokine networks, (3) regulation of apoptotic pathways, and (4) interaction with the complement system, collectively underpinning its critical regulatory roles (85). In oncology, SERPINA1 exhibits altered expression across cancer types, with significant heterogeneity among immune and molecular subtypes (86, 87). In lung cancer, AAT promotes malignant progression by inhibiting staurosporine-induced apoptosis/autophagy, emerging as a novel metastasis intervention target (88–90). Pancreatic cancer studies link elevated AAT levels to advanced TNM staging and independent poor prognosis (91). Conversely, AAT downregulation in breast cancer correlates with aggressive phenotypes, metastatic propensity, and worse outcomes (92, 93). SERPINA1 further associates with lymph node metastasis and immune cell infiltration in thyroid cancer (94). While in gliomas, it drives malignancy via anti-apoptotic mechanisms, with expression escalating from grade III to IV tumors (95, 96) and influencing prognosis and immune microenvironment in lower-grade (I/II) gliomas (97). Emerging evidence highlights its therapeutic potential in bladder cancer (98), osteosarcoma (99), and colon cancer (100), positioning SERPINA1 as a dual prognostic biomarker and actionable target. Future efforts must prioritize delineating its regulatory networks and advancing therapeutic validation to unlock precision oncology applications.

In our investigation, the four-gene prognostic signature (*MAOB, IGFBP2, SERPINA1* and *LGR6*) we identified demonstrates significant involvement in multifaceted oncogenic pathways, particularly exhibiting strong correlations with tumor proliferation, metastatic potential, and invasive behavior in WHO grade II/III gliomas. This molecular profile has emerged as a robust independent biomarker for predicting clinical outcomes in glioma patients. The ICBatlas analysis revealed the conserved bidirectional pattern (protective *MAOB/SERPINA1* vs. risk-enhancing *IGFBP2/LGR6*) of the four-gene signature as a pan-cancer theoretical framework for risk stratification. This finding, derived from cancer-type-level aggregation (12 cancer types), necessitates tissue-specific validation prior to clinical translation. To further substantiate these findings at the protein expression level, we performed IHC staining on clinical glioma specimens and adjacent non-tumorous tissues. All four proteins exhibited markedly elevated expression in tumor tissues, consistent with transcriptomic predictions and supporting their biological relevance in glioma progression.

However, several methodological and mechanistic limitations require prudent acknowledgment. Firstly, while our prognostic model demonstrated robust performance in the CGGA cohort, its validation across multi-institutional cohorts and diverse sequencing platforms (RNA-seq vs microarray) remains imperative. The retrospective design and single-center data source introduce potential selection bias that may impact the generalizability of findings. Secondly, our statistical approach for differential gene expression analysis (adjusted *p* < 0.05, |log_2_FC| > 1) might have excluded biologically relevant genes below significance thresholds, particularly those involved in compensatory pathways or exhibiting tissue-specific expression patterns. Thirdly, although bioinformatics analyses implicate these genes in oxidative phosphorylation (OXPHOS) pathways, their precise mechanistic roles in glioma metabolism and treatment resistance remain undetermined. Future studies should employ CRISPR-Cas9 gene editing combined with metabolic flux analysis to delineate their functional contributions. Furthermore, subtype C2 paradoxically exhibits high immune scores alongside poor prognosis. We hypothesize that this apparent contradiction arises because the elevated immune infiltration represents an enrichment of immunosuppressive M2-polarized macrophages and Tregs, not effector anti-tumor immune cells. Lastly, the clinical translatability of this signature necessitates prospective validation in controlled trials assessing its utility in guiding therapeutic decisions, particularly for patients undergoing metabolic-targeted therapies.

These critical next steps will be essential for translating these molecular insights into clinically actionable tools, potentially providing a groundwork for personalized therapeutic strategies in neuro-oncology. Subsequent investigations should also explore dynamic expression changes during malignant progression and therapeutic interventions through longitudinal liquid biopsy approaches.

## 4 Materials and methods

### 4.1 Data sources and pre-processing

Genes associated with oxidative phosphorylation (OXPHOS) were curated from the Molecular Signatures Database (MSigDB v7.0). The hallmark gene set HALLMARK_OXIDATIVE_PHOSPHORYLATION was selected to identify 200 OXPHOS-related genes, with manual curation to exclude non-metabolic genes and retain those functionally linked to mitochondrial energy metabolism.

Transcriptomic profiles and clinical follow-up data from WHO grade II/III glioma tissues were retrieved from The Cancer Genome Atlas (TCGA). The raw data underwent rigorous preprocessing: (1) Clinical data filtering: Samples lacking critical clinical endpoints (e.g., overall survival, recurrence status) were excluded to ensure prognostic relevance; (2) Gene annotation conversion: ENSEMBL gene identifiers (ENSG IDs) were mapped to official Gene Symbols using the HUGO Gene Nomenclature Committee (HGNC) database to standardize gene nomenclature; (3) Duplicate gene resolution: For genes with multiple ENSG IDs mapped to the same symbol, the entry exhibiting the highest expression value was retained to minimize technical bias and ensure data uniqueness. Post-processing, 512 high-quality samples from the TCGA-grade II/III gliomas cohort were retained for subsequent analysis.

RNA-Seq datasets of grade II/III gliomas were acquired from the Chinese Glioma Genome Atlas (CGGA). Preprocessing included: (1) Clinical completeness check: Samples without documented clinical follow-up (e.g., survival time, treatment response) were systematically removed; (2) Transcriptomic data integrity Validation: Samples with incomplete or corrupted expression profiles (e.g., sequencing failures, low read depth) were discarded. After stringent quality control, 420, 172, and 159 samples from three independent CGGA sub-cohorts (CGGA-693, CGGA-325, and CGGA-301) were retained, ensuring dataset robustness for cross-validation.

### 4.2 Non-negative matrix factorization-based consensus clustering for molecular subtypes identification

The TCGA RNA-seq profiles underwent rigorous preprocessing to prioritize biologically relevant features. First, genes exhibiting low variability (absolute log2-transformed fold change <1) or sparse expression (detected in <50% of samples) were filtered out. Prognostically significant OXPHOS genes were then identified through univariate Cox proportional hazards regression (*p <* 0.05). Consensus clustering was subsequently performed using ConsensusClusterPlus (v1.48.0; parameters:reps = 100, pItem = 0.8, pFeature = 1,distance = “spearman”) to delineate molecular subtypes. Subtype robustness was further validated via D2 clustering (partitioning around medoids algorithm) with Euclidean distance, achieving high concordance.

Differentially expressed genes (DEGs) between subtypes were identified using the limma package (|log2FC| >1, *p <* 0.05), followed by functional annotation via DAVID for Gene Ontology (GO) and KEGG pathway enrichment. Parallel gene set enrichment analysis (GSEA) was conducted using subtype-labeled expression profiles (C1 vs. C2) and the MSigDB hallmark gene sets (v7.0), with significant pathways defined by nominal *p <* 0.05 and FDR < 0.25.

### 4.3 Division of the datasets into training and validation subsets

The 512 samples from the TCGA datasets were randomly split into equal-sized training and validation sets using a 1:1 ratio through stratified random sampling. When selecting the optimal validation and training sets, the following criteria were strictly followed: (1) ensuring balanced distributions of patient gender, age, follow-up duration, and mortality rates between cohorts; (2) maintaining balanced inter-group quantities of binary classification samples after clustering analysis of gene expression profiles.

This rigorous approach yielded two 256 samples cohorts called training set and validation set, ensuring statistical similarity between the two groups. To achieve this, iterative resampling with propensity score matching was implemented to minimize covariate imbalances, while cluster-based stratification guaranteed comparable representation of molecular subtypes across both sets.

The final grouping satisfied Kolmogorov-Smirnov tests (p > 0.05) for continuous variables and χ²tests (p > 0.1) for categorical features, confirming the validity of the partitioning strategy.

### 4.4 Pan-cancer ICBatlas analysis

To investigate the prognostic relevance of immune checkpoint inhibitors (ICIs), we performed pan-cancer single-gene correlation analyses of *MAOB, IGFBP2, SERPINA1*, and *LGR6* based on transcriptomic data using the online platform ImmunoCheckDB (101) (web:https://smuonco.shinyapps.io/ImmunoCheckDB). Briefly, (1) representing cancer-type gene expression via median log2 (RSEM+1); (2) calculating Pearson’s and spearman’s *r* with median OS; (3) applying FDR-corrected t-tests; (4) visualizing relationships through scatterplots with LOESS regression curves, scaling point size to cohort sample size and shading 95% confidence intervals of the linear fit.

### 4.5 Lasso cox regression analysis

In order to refine the prognostic gene signature for grade II/III gliomas, we applied Lasso Cox regression—a shrinkage estimation method—to reduce dimensionality and enhance model interpretability and predictive accuracy. The Lasso (Least Absolute Shrinkage and Selection Operator) method introduces an L1-norm penalty function that selectively shrinks coefficients toward zero, effectively performing automated variable selection while addressing multicollinearity in high-dimensional data. This approach resolves critical challenges in regression analysis, including biased parameter estimation and overfitting.

Using the glmnet,in R package, we conducted Lasso Cox regression to analyze variable trajectories and optimize model performance. The regularization path was generated by iterating over a sequence of lambda (λ) values, which control the penalty strength. To identify the optimal λ, we implemented five-fold cross-validation, minimizing the partial likelihood deviance. The confidence interval analysis at each λ threshold revealed the stability of coefficient estimates, ultimately yielding a parsimonious model with 48 candidate genes.

### 4.6 Immunohistochemistry

Paraffin-embedded glioma and matched adjacent non-tumorous brain tissues were collected from grade II/III glioma patients who underwent surgical resection at The First Affiliated Hospital of Xinxiang Medical University, with pathological confirmation and informed consent under institutional ethical approval.

Tissue sections (4 μm) were deparaffinized in xylene, rehydrated through graded ethanol, and subjected to heat-induced antigen retrieval in sodium citrate buffer (pH 6.0) using a microwave. Endogenous peroxidase was blocked with 3% hydrogen peroxide for 10 min, followed by 10% goat serum for 30 min at room temperature to reduce non-specific binding. Sections were incubated overnight at 4°C with primary antibodies against MAOB (Thermo Fisher, PA5-79624), SERPINA1 (Proteintech, 82918-1-RR), LGR6 (Proteintech, 17658-1-AP), and IGFBP2 (Proteintech, 11065-3-AP), each at 1:100 dilution. After PBS washes, HRP-conjugated goat anti-rabbit IgG secondary antibody (Abcam, ab205718) was applied for 30 min at 37°C. Signals were developed using DAB (Beyotime, P0202) and counterstained with hematoxylin. Slides were dehydrated, mounted, and imaged using a light microscope (Nikon).

## Data Availability

The datasets analyzed in this study are derived from publicly available repositories, and the specific data sources are included in the article/**Supplementary Material**.
